# Psycho-social resilience, vulnerability and suicide prevention: impact evaluation of a mentoring approach to modify suicide risk for remote Indigenous Australian students at boarding school

**DOI:** 10.1186/s12889-016-2762-1

**Published:** 2016-02-01

**Authors:** Janya McCalman, Roxanne Bainbridge, Sandra Russo, Katrina Rutherford, Komla Tsey, Mark Wenitong, Anthony Shakeshaft, Chris Doran, Susan Jacups

**Affiliations:** 1 The Cairns Institute, James Cook University, PO Box 6811, Cairns, QLD 4870 Australia; 2 Transition Support Service, Queensland Department of Education, Training and Employment, P O Box 2268, Cairns, QLD 4870 Australia; 3 Apunipima Cape York Health Council, PO box 12045, Westcourt, QLD 4870 Australia; 4 National Drug and Alcohol Research Centre, University of NSW, 22-32 King St, Randwick, NSW 2031 Australia; 5 CQUniversity, 160 Ann Street, Brisbane, QLD 4000 Australia; 6 School of Human Health and Social Sciences, CQUniversity Australia, Cairns Square, Level 3, Corner Abbott and Shields Streets, Cairns, QLD 4870 Australia

**Keywords:** Resilience, Suicide prevention, Wellbeing, Aboriginal, Torres Strait Islander, Remote, School students, Boarding school, Mentoring

## Abstract

**Background:**

The proposed study was developed in response to increased suicide risk identified in Aboriginal and Torres Strait Islander students who are compelled to attend boarding schools across Queensland when there is no secondary schooling provision in their remote home communities. It will investigate the impact of a multicomponent mentoring intervention to increase levels of psychosocial resilience. We aim to test the null hypothesis that students’ resilience is not positively influenced by the intervention. The 5-year project was funded by the Australian National Health and Medical Research Council from December 2014.

**Methods/Design:**

An integrated mixed methods approach will be adopted; each component iteratively informing the other. Using an interrupted time series design, the primary research methods are quantitative: 1) assessment of change in students’ resilience, educational outcomes and suicide risk; and 2) calculation of costs of the intervention. Secondary methods are qualitative: 3) a grounded theoretical model of the process of enhancing students’ psychosocial resilience to protect against suicide. Additionally, there is a tertiary focus on capacity development: more experienced researchers in the team will provide research mentorship to less experienced researchers through regular meetings; while Indigenous team members provide cultural mentorship in research practices to non-Indigenous members.

**Discussion:**

Australia’s suicide prevention policy is progressive but a strong service delivery model is lacking, particularly for Indigenous peoples. The proposed research will potentially improve students’ levels of resilience to mitigate against suicide risk. Additionally, it could reduce the economic and social costs of Indigenous youth suicide by obtaining agreement on what is good suicide prevention practice for remote Indigenous students who transition to boarding schools for education, and identifying the benefits-costs of an evidence-based multi-component mentoring intervention to improve resilience.

## Background

During their most vulnerable developmental life phases, some 515 Aboriginal and Torres Strait Islander students from Australia’s remote regions of Cape York and Palm Island are compelled to transition away from home to boarding schools. They are a subset of the approximately 4165 Aboriginal and Torres Strait Islander secondary school students (11–18 years) from remote communities across Australia who accessed the means tested Schools Fees Allowance (Boarding) Supplement administered by the Aboriginal and Torres Strait Islander Student Assistance Scheme (ABSTUDY). These students lack educational options, and hence are obligated to participate in the trend toward boarding schools in Aboriginal and Torres Strait Islander education [1, 2]. Transitions to boarding schools involve far more than simply negotiating the logistics of shifting from one school to another. Students in transition face major life changes: changes in residence; cultures, including language; autonomy; educational standards; roles, responsibilities and expectations; parental influence; personal freedom; and relationships; and are often confronted with institutional discrimination and racism [3, 4]. School transitions also coincide with physiological changes from childhood to adolescence and emerging adulthood, and are compounded by associated increasing peer pressure, heightened participation in risky health behaviours such as alcohol and drug consumption and sexual activity, and increased risk of depression [5, 6]. Additional transitions then come when students return to their home communities and/or move from school into employment or further study [1]. These transitional stressors alone clearly place these young people in a heightened suicide risk category. Yet, there is little research about the unique circumstances that exacerbate their suicide risk – neither students’ stress in managing transitions, other demographic and socio-economic factors and transgenerational trauma experienced by many Aboriginal and Torres Strait Islander youth, nor the unfamiliar customs and routines of boarding school environments or changes in relationships with families and communities of origin [1, 6]. Transitional stresses affect students differently, and the complexity of the multiple transitions faced by remote-dwelling youth suggests a need for enhanced support in transition processes. Unfortunately, we do not know what works to build student resilience to deal with such increased vulnerability to suicide and other risks.

### Aboriginal and Torres Strait Islander suicide: a snapshot

Aboriginal and Torres Strait Islander young people reportedly experience the highest risk of suicide in Australia - amongst men between 25 and 29 years, and, amongst women between 20 and 24 years [7, 8]. Alarmingly, Aboriginal and Torres Strait Islander children under 15 years have a suicide rate of 7 times their non-Indigenous peers; with 15–24 year olds having a rate 3.6 times the same peers [9]. In small remote communities, these high suicide rates have devastating community-wide impacts. Contributing to suicide is the high exposure of Aboriginal and Torres Strait Islander youth to risk factors. A social survey of Aboriginal and Torres Strait Islander young people aged 15–24 reported that while 69 % of youth experience low to moderate levels of psychological distress (79 % male and 65 % females); a further 29 % experience high or very high levels of distress (20 % males and 35 % females) [10]. Importantly, the survey found that those youth with lower psychological distress were more likely to be studying and have family members or friends outside their household in whom they could confide [10]. Youth who are studying were also less likely to partake in risky health behaviours, such as substance abuse that reportedly surrounds suicide risk [11]. These conclusions from national surveys are consistent with findings of studies in north Queensland discrete Aboriginal and Torres Strait Islander communities. A survey of young people from one such community found that binge drinking stemmed primarily from boredom, defined by young people as “a deeper lack of purpose, engagement or meaning in life” [12]. Research participants requested mentoring into education and employment as a potentially effective strategy to counter boredom and thus reduce alcohol-related harm in young people [12]. Inferences drawn from these data, point to several implications for investigating and developing effective suicide prevention interventions: 1) a critical need to intervene during the earlier adolescent years; 2) the importance of engaging and retaining young people in study or other meaningful occupations; and 3) the value of providing opportunities for young people to engage with significant others in their lives.

### Risk and protective factors

The circumstances surrounding Aboriginal and Torres Strait Islander Australian suicide risk differ from that of non-Indigenous populations to include discrete factors related to the legacies of colonisation. Thus exposure to significant historical and contemporary adversity has adversely affected Aboriginal and Torres Strait Islanders’ wellbeing and resilience: “both the capacity of individuals to navigate their way to the psychological, social, cultural, and physical resources that sustain their well-being, and their capacity individually and collectively to negotiate for these resources to be provided in culturally meaningful ways” [13]. Distal and proximal determinants of suicide risk include: 1) demographic and social/economic factors (e.g. poverty, unemployment, reduced service access, homelessness, remoteness); 2) personal history of risk factors (e.g. trauma or grief from discrimination, removal of children, premature deaths of community members and loss of cultural identity, sexual or physical abuse or neglect, physical and mental illness, high rates of interpersonal violence, history of self-harm, substance abuse, juvenile detention, police custody); and 3) current personal risk factors (e.g. cultural or religious conflicts, no social support networks, at risk mental status, recent interpersonal crisis, loss or trauma, family breakdown, child custody issues, influence of alcohol or drugs, difficulty accessing help; financial difficulties or unemployment, legal prosecution, illness) [14]. These risks cumulate and feed into each other [15].

Along with some evidence for cultural continuity [16, 17], self-determination and community control [17], high levels of mental health and wellbeing and social support are cited as protective against suicide risk [18]. Support programs that strengthen family and community support networks, while simultaneously promoting the ability of young people to cope with daily stresses have been identified as important resources in treatment and preventative programs [8]. Programs that are culturally competent, have a high level of Aboriginal and Torres Strait Islander ownership and community support, and, promote social, emotional, cultural and spiritual wellbeing can be effective in suicide prevention [8, 18]. Studies have also found that improving problem solving, coping with stress, and increasing resilience enhance protective factors [19, 20].

### What works in suicide prevention?

There is a lack of compelling empirical evidence regarding interventions for Aboriginal and Torres Strait Islander Australian suicide prevention. The findings of our recent systematic review of suicide intervention literature targeting Indigenous peoples in Australia, United States, Canada and New Zealand showed that there are negligible systematic and controlled studies evaluating the efficacy of interventions in global Indigenous populations generally, or of suicidal adolescents [21]; and thus it is difficult to ascertain what works best. The review found only three publications that evaluated interventions targeting Aboriginal and Torres Strait Islander Australians. Two described gatekeeper training - outcomes showed significant improvements pre-post training in knowledge and confidence in how to identify individuals at suicide risk [22, 23]; and the third was a social and emotional empowerment education program, the Family Wellbeing Program (FWB) [24]. Pilot projects, wherein the Aboriginal-developed FWB was delivered as a suicide prevention program, found it acceptable as a protective strategy against suicide risk. Participants perceived that the program helped them work through issues and exert greater control over their social and emotional wellbeing [24–27]. FWB is a generic approach and can be tailored to suit the audience – examples include its adaptation for school students in grades six and seven [28]. Improvements in perceptions of personal empowerment included self-worth, resilience, problem solving ability, and belief in the mutability of the social environment [24, 29]. These findings suggest the need for an impact evaluation of FWB in promoting resilience against suicide.

Much of the broader international literature on suicide has focused on the determinants of suicide risk, and pursued explanations of the relationships between these and suicide outcomes. Despite limited evidence, this literature highlights multi-component suicide prevention approaches for young people, including screening to identify those at risk of suicide [21, 30]. Three types of suicide prevention programs have been identified: 1) those that build resilience; 2) crisis intervention programs; and 3) post-intervention programs [31]. The proposed study is concerned with resilience-strengthening. For Aboriginal and Torres Strait Islander students, having the resilience to make healthy adjustments in times of high vulnerability is vital to maintaining their wellbeing. While prevention should focus on both the risk and protective factors [32], resilience theory has increasingly explored understandings of why some youth who experience adversity are able to avoid harmful, self-destructive, or antisocial behaviours, mental disorders, and threats to their physical wellbeing. Social ecological models theorise that resilience is not just about the personal qualities of the student, but how well their social and physical environment (including the school, family, and community) facilitates access to internal and external resources such as healthy relationships, a powerful identity, social justice, material needs like food and education, and a sense of belonging, life purpose and spirituality [33]. Student participation in processes that support them to navigate to such resources and negotiate for them to be provided in meaningful, culturally relevant ways is more likely to translate into positive wellbeing and educational outcomes [34]. Like the proposed study, studies of resilience in relation to suicide offer encouraging approaches that move toward constructive behaviours and life-enhancing competencies in contrast to research concerned with those developmental deficits and pathological approaches that have saturated Indigenous research in the past and failed to produce social change. School-based mental health promotion programs that promote resilience among young people have not reported significant benefits for rates of suicide ideation or help-seeking, but have demonstrated increased knowledge, improved attitudes to mental illness and suicide, lowered suicide attempt rates, and enhanced adaptive attitudes about depression and suicide post-intervention [8, 20, 28, 34].

An associated strategy, mentoring, has been demonstrated as a firm predictor of resilience and empowerment for young people [35]. When youth have a trusting relationship with a caring adult, negative effects from their environment reduce and positive outcomes generally occur [36]. Studies in Australia e.g. [37], and beyond, show that young people who feel connected to a supportive adult engage in less health-risk and other problem behaviours (including suicide and self-harm), and improve in youth competencies [38–41]. Benefits are particularly apparent for those facing more complex environmental risks and challenges with stressors and adversity [42]. Youth mentoring can improve social, emotional, educational and economic outcomes for young people: including academic achievement, increased school attendance, positive school behaviours and attitudes, greater wellbeing, connectedness to others, improved social relationships, encourage active citizenry and changed life course [35–37]. Our recent review on Aboriginal and Torres Strait Islander Australian mentoring [43], described a growing body of research demonstrating that mentoring can have powerful and lasting positive effects in improving the behavioural, academic and vocational outcomes for at-risk youth; themselves protective factors for suicide risk. Most effective are culturally-tailored, long-term, formal, one-on-one, integrated mentoring models that account for mentor competence and support and which are integrated into broader support services and programs, producing a greater level of positive change [43, 44]. However, while mentoring approaches are promising, their effectiveness in promoting resilience against suicide risks has not been rigorously tested.

### Study aims

The 5 year study was developed in partnership with Education Queensland’s Transition Support Service (TSS) in response to increased suicide risk identified in transitioning students. It was funded by the National Health and Medical Research Council from December 2014. The study will investigate the impact of an enhanced multicomponent mentoring intervention to increase levels of psychosocial resilience among the 515 remote Aboriginal and Torres Strait Islander students from Cape York and Palm Island, who are compelled to relocate to boarding schools across Queensland when there is no secondary schooling provision in their home communities. We aim to test the null hypothesis that students’ resilience is not positively influenced by the multicomponent mentoring suicide prevention intervention. The objectives of this study are to:

1. Enhance the existing TSS case management approach by supporting a multi-component mentoring intervention for staff and assessing its impact;
2. Identify and apply valid and reliable quantitative outcome measures to assess the impact of the enhanced resilience-based service delivery model for Aboriginal and Torres Strait Islander students using an interrupted time series design;
3. Apply cost analysis methods to calculate the economic costs of the intervention; and
4. Apply grounded theory methods to theorise and explain the process by which psychosocial resilience was enhanced to protect against suicide for Aboriginal and Torres Strait Islander students, including the contextual factors.

## Methods/Design

### Design of the study

This is not stand-alone investigator-driven research, but rather an excellent example of researchers responding to the needs expressed by Aboriginal and Torres Strait Islander communities for effective programs to empower and promote wellbeing. The proposed study builds on extensive existing networks and research partnerships developed and supported over ten years. A strength-based participatory approach founded on social constructivism will be applied [45, 46]. Participatory research approaches offer opportunities for researchers and participants to produce change by working together in more equitable relationships, and this will be employed in ways that facilitate Aboriginal and Torres Strait Islander aspirations of autonomy, self-determination and empowerment for individuals, families and communities. The investigator team has successfully collaborated using the model of Community-Based Participatory Research (CBPR) espoused by Minkler and Wallerstein [47] with Aboriginal and Torres Strait Islander communities, schools, men and women’s support groups and to promote organisational change in community-controlled organisations over many years [12, 24, 25, 28, 48]; this has established CBPR as an acceptable and feasible research approach that is highly engaging for Aboriginal and Torres Strait Islander people [48]. CBPR promotes sustainability, mutual trust and respect in the relationship with Aboriginal and Torres Strait Islander people, partnerships, ownership and empowerment in the process, and benefits to the research population [48]. As demonstrated in previous studies, Aboriginal and Torres Strait Islander participation at all levels of the research will ensure Aboriginal and Torres Strait Islander capacity-development, engagement and ownership of the research, while collaborative and participatory approaches will enable immediate translation of the research results into practice [12, 24, 48]. Research translation through bi-annual knowledge sharing forums with Education Queensland, partner organisations and a project youth sub-committee is also a routine part of the research plan.

Using a mixed methods design, complementary quantitative and qualitative data will be combined to deliver a robust and nuanced picture of strengthening psychosocial resilience as a suicide prevention intervention. As the key outcomes of interest, the levels of change in students’ resilience, educational outcomes and suicide risk will be tested using an interrupted time series design. The costs of mentoring as an approach for youth suicide will also be calculated. Concurrently, a theoretical explanation of the process by which the psychosocial resilience and education outcomes of students were enhanced (or not) through the intervention will be established qualitatively using grounded theory methods. Qualitative and quantitative methods will be triangulated by comparing how the qualitative data find support and confirmation (or otherwise) in the quantitative results, without transformation of either data [49].

### The setting

**Fig. 1:**
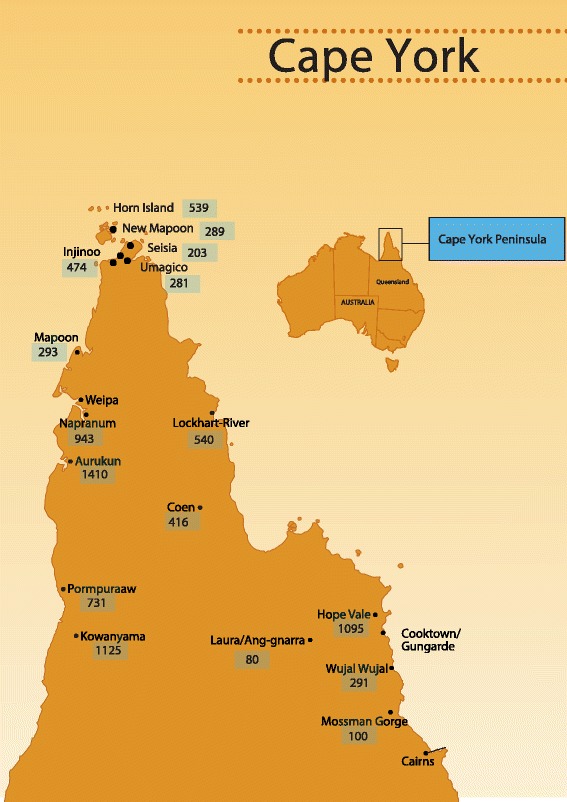
Cape York communities

TSS uses a case management approach based on a skilled helper mentoring model to support students from Palm Island and Cape York communities (Fig. 1): Weipa, Old Mapoon, Napranum, Aurukun, Pormpuraaw, Kowanyama, Lockhart River, Coen, Hope Vale, Cooktown, Laura, Rossville and Wujal Wujal to manage the transition challenges and develop opportunities that lead to Year 12 secondary leaving certificate attainment (or equivalent) and pathways beyond Year 12. TSS also engages with families, the 34 destination boarding schools from Weipa in the north of Queensland to Toowoomba in the south, and partner services (e.g. Families Responsibility Commission, Department of Communities, Juvenile Justice, Apunipima Cape York Health Council) that impact on students’ adjustment, orientation and ongoing stay at boarding school. However, issues for many students arise from the inadequacy of academic and social and emotional wellbeing preparation during primary school years and challenges in adjustment. These challenges resulted in 51 % of the 1026 Cape York students supported by TSS between 2008-March 2013 becoming de-enrolled and not returning to boarding school to complete their secondary schooling (Pers. Comm. AI Russo, 10/10/2013). We do not know the precise levels of suicide or self-harm experienced by these students as there is no reliable data, but anecdotal evidence strongly suggests high levels of vulnerability e.g. since 2008, there has been three cases of suicide completion, 4 known hospitalisations for self-harm and ongoing concern about the normalisation of suicide ideation among some members of the cohort.

Part of the project’s significance is developing good baseline data, which is itself a research outcome and will provide the basis for future work. Additionally, the limitations of the current case management approach, including its reactive nature rather than focus on the long-term goals and provision of structured support for students and the lack of regular professional mentoring/ debriefing support for staff, suggest a need for explicit engagement with suicide prevention and best practice mentoring principles.

### The participants

We will work with TSS staff as mentors (24) and students as mentees (515) (*n* = 539). The mentors include all management, Transition Support Officers, Community Support Officers and Youth Mentors across the three streams of TSS: 1) transition from the primary to secondary phase of schooling; 2) engagement with secondary schooling and transition to work, training or further education at completion of Year 12; and 3) re-engagement with learning, training or employment pathways when enrolment at boarding schools is not an option. Mentees include the entire cohort of students assisted by the TSS: in 2015, 114 Year 7 students and families; 261 secondary school-aged students at boarding schools; and 140 students who have become de-enrolled from boarding schools and who have returned to their communities.

### Ethics

The study proposal was submitted to and approved by the James Cook University (H5964, H6295) and Education Queensland (550/27/1646) Ethics Committees. Informed consent processes were approved for students, parents and departmental staff members. These comprised written information sheets and appropriate (to culture and maturity) oral descriptors of the project, survey/interview processes, rights to participate or not and withdraw without penalty, confidentiality and security of data, and processes for addressing risks or concerns associated with the research, including contact numbers. Risk identification and risk management strategies incorporated: 1) training and support for mentors to conduct the screening and engage and support students at risk to access the 24-hour Suicide Call Back Service and/or specialist services; and 2) mentor support from CI/AI clinicians to develop and implement individual referral pathways for each identified student at risk as part of their mentoring plans. Only researchers doing the analysis and the relevant mentor will have access to the identity of the students at risk. Aggregated data supporting the study’s findings will be lodged, upon completion of data collection, through appropriate data repositories and by contacting the lead author of this paper.

### Interventions and analysis

A complementary suite of methods correlates with each of the study objectives.

### Objective 1: Enhance the existing TSS case management approach by supporting a multi-component mentoring intervention for staff, and assessing its impact

Based on the evidence and feasibility of strengthening the current case management/mentoring approach of TSS, the research partnership identified the need for a multi-component suicide prevention mentoring approach to: 1) enhance the current workforce competencies of TSS staff through explicit empowerment and suicide awareness training (a tailored FWB and gate-keeper training); and 2) provide regular follow up support for mentors using reflective CBPR frameworks to continuously improve the mentoring approach and strengthen the capacity of TSS staff to mentor students (mentees) to modify the risk and protective factors for suicide.

*First*, we will refine a multicomponent mentoring suicide prevention training package for delivery to mentors. The investigator team has previously piloted the FWB as a single intervention and documented its acceptability, feasibility and outcomes. We will value-add by bolstering FWB delivery with gatekeeper training (Aboriginal and Torres Strait Islander Mental Health First Aid), resilience and mentoring training.

*Second*, The components of the training will be confirmed in collaboration with TSS but are likely to include: **1) Training in FWB 12 days**: Training will include FWB modules for examining and understanding human qualities, human needs, relationships, conflict and the process of change, emotions, crisis, beliefs and attitudes, family violence and loss and grief; **2) Gatekeeper training 2 days**: including suicide risk assessment and management; 3) **Resilience training 1 day**: including the nine things that young people need and how to build a resilience approach; and **3) Mentor training 0.5 days**: guidance for implementation of the mentoring approach to students to ensure implementation fidelity. With consent, baseline data, post-training and 6 month post-training data will be collected for TSS staff from surveys of wellbeing and confidence to implement the mentoring approach.

We will measure mentor changes in: 1) empowerment (benefits, stimulation, challenge and reward), and 2) confidence (understanding roles, commitment, and relationships of trust and respect) which are critical for effective mentoring relationships [36]. Measures are likely to include the Growth Empowerment Measure [50] and Participatory, Results-oriented, Self-evaluation (PROSE) tools [51]. Mentor changes in empowerment and confidence will also be measured using a tailored survey at baseline, post-training and at 6 months post-training.

*Third*, trained TSS staff will be engaged through CBPR critical reflexive sessions to define a resilience-based model of student support. The details of the model will be worked out in partnership with TSS, but it will be incorporate student workshops, learning plans, resilience strengthening and post-school aspirations. Opportunities for one-on-one and/or group mentoring will be identified. CBPR critical reflexive sessions will be continued over 30 months (continuous quality improvement in practice). Based on experience, 30 months is more than adequate to determine improvements. Feeding into CBPR processes will be baseline routinely collected and screening survey data (Objective 2), and evidence from the suicide prevention and mentoring literatures. Established questions will guide CBPR processes: how are we going; what is working, what is not; are we getting our fair share of resources relative to need; who is benefiting; who is missing out; what can be done to reach those people; how can we improve our situation. CPBR processes will be carefully documented. The continual improvement of the evidence-informed mentoring approach will contribute to shifting the focus of the current TSS case management approach from one of relative distance and constraints to a formalised mentoring relationship that provides support and capacity enhancement to students to negotiate their day to day challenges of life without being overwhelmed by them.

### Objective 2: Identify and apply valid and reliable quantitative outcome measures to assess the impact of the enhanced resilience-based service delivery model for Aboriginal and Torres Strait Islander students using an interrupted time series design

**Fig. 2:**
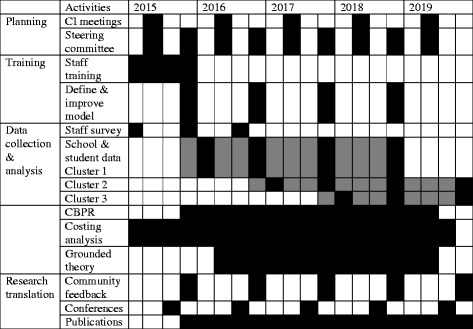
Research deliverables

An interrupted time series design will be applied to evaluate the effects of the resilience-based training and redefined model of student support in modifying mentees’ resilience, educational outcomes, and suicidal risk. The research design provides practicality and rigour by staggering implementation of the mentoring approach, enabling each group to act as a comparison group for itself and the other group [52]. This design is endorsed by the Cochrane Effective Practice and Organization of Care Group as an alternative to randomised control trials. The design is based on randomisation of the primary and secondary schools and groups of de-enrolled students supported by TSS staff into three clusters where students supported by TSS will receive the resilience-based model of student support starting 2016; 2017 and 2018 (Fig. 2).

Selection criteria for the inclusion of schools will be collaboratively negotiated with TSS. The criteria will likely include: 1) having an enrolment of at least 10 TSS-supported students at the school and 2) being representative of the five TSS regions. We will also collect data for students from routinely available education indicators such as school attendance, retention. We will repeat the process with the second and third clusters of schools from 2017 to 2018.

We will assess the impact of the suicide prevention intervention on students’ resilience using: 1) routinely collected school outcomes data; and 2) screening survey data for the aggregated student cohort. Data routinely collected by Education Queensland (school attendance, retention and post-school destination) reflects suicide protective factors. Survey measures will be refined by the project steering group, but we anticipate a tailored instrument incorporating: resilience (individual capacities/ resources, relationships with primary caregivers and contextual factors that facilitate a sense of belonging), accompanied by the Kessler 5 psychological distress scale and suicidal risk factors, supplemented by questions assessing frequency of happy and angry feelings. Resilience will be measured using the Child and Youth Resilience Measure (CYRM) which has been validated internationally [53]. The suicide risk assessment questions are standard screening questions which were identified by a local Indigenous medical practitioner and CI as most relevant for Cape York students. The screening instrument will also include questions about services of which students are aware and access and transition strategies that they perceive to work. Using agreed measures, an online student screening survey will be integrated into TSS processes for student placements into boarding schools and administered using iPads to students in each cluster at the start of the 2016, 2017 and 2018 academic years.

Taking account of an expected drop-out rate of senior students who complete school; and the high likelihood of tracing students due to the close linkages between TSS staff with students and their families, we expect a follow up rate of 80 % at mid-point (*n* = 412) and 80 % at post-intervention (*n* = 330). These sample size calculations are based on population measures and study design. The primary outcome measures will be resilience (measured using the CYRM), and school participation (measured through routine data and including a composite indicator comprising uptake of placements at boarding schools at start of grade 7; uptake of the first scheduled flights to boarding schools each term; and a reduction in the rate of suspension for students who re-engage for issues that relate to not staying enrolled). Based on previously conducted studies using ANOVA for analysis, we assumed a 10 % effect size [53]. This is very conservative. We assumed a standard deviation of (0.3). Sample size calculations for an alpha level of 5 %, with power 80 %, *n* = 68 students per group, which will sufficiently enable statistical testing of the hypothesised differences. Since we had planned to sample *n* = 172 mentored students per group, about three times the sample size required, we are confident that our sample will be adequate for additional group-specific stratified analyses. The identified results of the routinely collected and screening survey data will be fed back to TSS staff to tailor the development of mentees’ aspirational plans. Following the baseline screening survey, the same instrument will be implemented at the end of the academic year and upon completion of the following two academic years.

A fixed cohort approach is planned [54] with the above-mentioned outcome measures for the 172 mentored students in group 1 compared pre- and post-intervention and to measures for the 172 students in groups 2 and 3. Separate statistical analyses will be undertaken for each outcome for each group using the statistical software program, STATA. The biennial proportion of students with each outcome of interest will be considered as a continuous measure for the analyses. Evaluation of the intervention effect in an interrupted time series design involves fitting a disjointed, segmented, linear regression model for the proportion of individuals with the outcome of interest over time, separately for each group. The models will include separate intercepts and slopes for both pre- and post-intervention (the two “segments”) and a term for site. For the intercepts, an intervention effect will be identified by a change or ‘jump’, in the primary outcome from pre- to post-intervention. For the slopes, an intervention effect will identified by a change in trend from pre- to post-intervention [55]. Examination of the magnitude of these coefficients will determine whether they are statistically significant.

### Objective 3: To apply cost analyses methods to estimate the economic costs of the intervention

The costs of implementing the intervention in monetary terms will be established using costing analysis methods, previously applied to cost a FWB intervention [56]. Previously used instruments will be utilised to cost and appropriately analyse the full spectrum of resources utilised in the interventions. International guidelines for estimating economic costs will be adhered to. Complementing information from routinely collected data, standardised self-report forms will be used to record the characteristics of time expended by staff (akin to timesheets). Activity codes will be assigned to identify the type of contact that occurred. Tangible items (supplies, consumables) will be monitored and recorded.

### Objective 4: Apply grounded theory methods to theorise and explain the process by which psychosocial resilience was enhanced to protect against suicide for Aboriginal and Torres Strait Islander students, including the contextual factors

The documentation of CPBR processes’ and observational data (described in Objective 1) will be combined with data from interviews and focus groups to capture the totality and richness of relational experiences evidenced in the mentoring approach for promoting resilience against suicide risk. We will engage a theoretical sample of mentors, mentees, families and Elders, community members and partner service providers. Theoretical sampling is a central and recurrent part of grounded theorising in generating and developing theoretical ideas. At various times researchers ask what settings, events, people etc. are useful investigating next in order to develop aspects of the emerging theory. It is guided by, and helps generate the theoretical sensitivity necessary [57]. Identification of the sample will be guided by TSS and community partners. Participation will be consensual; for students, parental approval will be sought. For young Aboriginal and Torres Strait Islander people, informal yarning groups work well vis-à-vis one-on-one interviews [58]. We will use outsider-witnessing techniques (story-sharing through technology with others outside the immediate environment) to encourage participation [59]. Interviews will take approximately 45 min and be conducted face-to-face at a negotiated place of the participant’s choice. Process assessment questions will guide interviews, covering issues such as the school, community and mentoring environments and processes at the initiation of the proposed research; other community events/issues impacting on individuals’ goals and objectives; the degree to which changes in indicators can be ascribed to TSS actions versus other factors; mentors’ perceptions of confidence and capacity to implement the suicide prevention mentoring intervention; and diverse views about mentees’ participation in, satisfaction with, and perceptions of personal change as a result of the mentoring activities; and what else needs to occur. These will be recorded with consent; de-identified, transcribed and fed back to TSS.

Interview transcripts, records of consultations, CBPR processes and observation data, published papers, reports and other relevant literature will be analysed using grounded theory methods in an ‘all is data’ approach [57]. Grounded theory is suited to conducting exploratory research, especially in areas like resilience promotion against suicide risk and the relationships between wellbeing, resilience and education, which lack an evidence base [12, 60–62]. Grounded theory will be used to structure a theoretical model that maps the pathways linking mentoring strategies and activities with resilience enhancement and education outcomes for Aboriginal and Torres Strait Islander students, as well as the contexts and conditions under which it develops; the actions and strategies manifest in the process; and the consequences of those actions. Grounded theory emergence, testing and modification in the light of new data will occur in accord with Glaser’s (1978) causal-consequence model [57]. Modelling will assist clarifications of process issues such as how research evidence can best facilitate TSS in achieving goals and effectiveness of mentoring strategies in supporting outcomes.

Expected outcomes are: an evidence informed mentoring program as a new service delivery model that integrates a resilience component into case management of remote area students transitioning to boarding schools that could be sustained in Queensland and adapted across Australia, changes in levels of resilience and education outcomes before and after, a grounded theoretical model, and assessment of social return on investment from Aboriginal and Torres Strait Islander youth mentoring.

## Discussion

Australia’s suicide prevention policy is progressive but a strong service delivery model is lacking, particularly for Aboriginal and Torres Strait Islander peoples [63, 64]. The proposed research will potentially reduce youth suicide and the social and economic costs of suicide by: 1) obtaining agreement on what is good suicide prevention practice for remote Aboriginal and Torres Strait Islander students who transition to boarding schools for education; and 2) identifying the benefits-costs of an effective evidence-based youth suicide mentoring prevention intervention. The research addresses the findings of multiple Indigenous-specific literature reviews, that there is currently an over-representation of descriptive research in the peer-reviewed published literature and insufficient impact/outcome evaluation research [65, 66]. The research design incorporates innovation and adaptability over the study period to contribute evidence on impact; i.e. evidence on whether a specific program/intervention actually works, an area notoriously under-researched in Aboriginal and Torres Strait Islander communities where too often only formative/process evaluation is conducted. Building an evidence base for Aboriginal and Torres Strait Islander-developed programs such as FWB demonstrates principles of equity and access, as intervention research can more appropriately respond to cultural and social aspects unique to Aboriginal and Torres Strait Islander individuals, families and communities. Improvements in young people’s resilience will contribute to wellbeing generally and translates to increased human and social capital, which manifests in areas such as reduced health and social risk and improved education/training, employment and other social participation [64]. Outcomes will thus strengthen cultural identity and pride as well as the aspirations/potential of Aboriginal and Torres Strait Islander Australians to contribute to the wider society. Economic benefit also flows from reduced Government investment in health and socio-economic risk.

The knowledge emanating from this project will substantiate and provide guidance on effective, acceptable and practical strategies to implement evidence-based interventions. In particular, the study will develop a new service model for the 4165 high risk Aboriginal and Torres Strait Islander Australian students from remote and very remote communities; the majority of whom transition to boarding schools for secondary education. The implications of the research will also have broader applicability for informing resilience strategies to prevent suicide risk in other high risk groups of students. The research will also determine the effectiveness of mentoring as a suicide prevention approach for all Aboriginal and Torres Strait Islander students. For Aboriginal and Torres Strait Islander people, the main benefit lies with the survival and protection of people and culture [15, 67]; specifically, enhanced knowledge about how to protect their young people through complex transition processes, as well as increased access to best evidence suicide prevention. The research is designed collaboratively such that at a minimum, its benefits will be sustained within Education Queensland’s TSS. For Education Queensland, the pragmatic outcome will be a tailored and sustainable evidence-informed multicomponent mentoring suicide prevention service model and unique evidence of the benefits-costs of suicide prevention that is practically relevant for enhancing efforts to build students’ resilience against suicide risk. For key community and broader stakeholders, biannual knowledge sharing forums will be used to translate the broader findings of the research and the service model to influence the protective factors for suicide prevention policy more generally for Aboriginal and Torres Strait Islander young people. Community stakeholders include extended family members and friends and community, regional and national partner organisations and networks. Research findings will also be translated through innovative project governance structures, Lowitja Institute Roundtable Forums and other health, education and international fora. For governments, knowledge generated will assist in identifying which suicide prevention strategies ought to be funded on a recurrent basis to facilitate more cost-effective rollout of best-evidenced suicide prevention interventions for Aboriginal and Torres Strait Islander young people. Through these media, we will provide clear advice to policy makers on the potential health, social and economic returns to Australian society from investments in evidence-informed suicide prevention and ensure that information flowing from the project is likely to be translated into outcomes that have an impact through changes in policy and practice.
